# Analysis of Thrombophilia Test Ordering Practices at an Academic Center: A Proposal for Appropriate Testing to Reduce Harm and Cost

**DOI:** 10.1371/journal.pone.0155326

**Published:** 2016-05-13

**Authors:** Yu-Min Shen, Judy Tsai, Evelyn Taiwo, Chakri Gavva, Sean G. Yates, Vivek Patel, Eugene Frenkel, Ravi Sarode

**Affiliations:** 1 Department of Internal Medicine, University of Texas Southwestern Medical Center, Dallas, Texas, United States of America; 2 Department of Pathology, University of Texas Southwestern Medical Center, Dallas, Texas, United States of America; Maastricht University Medical Center, NETHERLANDS

## Abstract

Ideally, thrombophilia testing should be tailored to the type of thrombotic event without the influence of anticoagulation therapy or acute phase effects which can give false positive results that may result in long term anticoagulation. However, thrombophilia testing is often performed routinely in unselected patients. We analyzed all consecutive thrombophilia testing orders during the months of October and November 2009 at an academic teaching institution. Information was extracted from electronic medical records for the following: indication, timing, comprehensiveness of tests, anticoagulation therapy at the time of testing, and confirmatory repeat testing, if any. Based on the findings of this analysis, we established local guidelines in May 2013 for appropriate thrombophilia testing, primarily to prevent testing during the acute thrombotic event or while the patient is on anticoagulation. We then evaluated ordering practices 22 months after guideline implementation. One hundred seventy-three patients were included in the study. Only 34% (58/173) had appropriate indications (unprovoked venous or arterial thrombosis or pregnancy losses). 51% (61/119) with an index clinical event were tested within one week of the event. Although 46% (79/173) were found to have abnormal results, only 46% of these had the abnormal tests repeated for confirmation with 54% potentially carrying a wrong diagnosis with long term anticoagulation. Twenty-two months after guideline implementation, there was an 84% reduction in ordered tests. Thus, this study revealed that a significant proportion of thrombophilia testing was inappropriately performed. We implemented local guidelines for thrombophilia testing for clinicians, resulting in a reduction in healthcare costs and improved patient care.

## Introduction

The clinical impact of venous thromboembolism (VTE) has increased significantly over the past decades. The incidence of a first episode of VTE, in the form of deep venous thrombosis (DVT) or pulmonary embolism (PE), or both, is approximately 1–2 per 1000 person-years[1,2]. Despite widespread use of prophylactic regimens, VTE remains a leading cause of preventable death among hospitalized patients[3]. Accordingly, identification of populations at risk for venous thrombosis has become a priority, and the search for thrombophilia markers has grown steadily since the discovery of antithrombin (AT) deficiency and dysfibrinogenemia in 1965[4,5].

Inherited risk factors for venous thrombosis include deficiencies of the natural anticoagulants AT, protein C (PC)[6], and protein S (PS)[7]. Patients may also possess genetic polymorphisms such as factor V Leiden (FVL) [8], prothrombin G20210A mutation (PGM)[9], or elevated levels of factor VIII (FVIII) [10]. Generally, patients with congenital thrombophilia develop VTEs without provocation or after a trivial insult at a relatively young age (<50 years) [11,12]. The most commonly encountered acquired thrombophilia is antiphospholipid antibody syndrome [13]. Venous thrombosis may rarely develop as a complication of hematopoietic stem cell disorders such as myeloproliferative neoplasms and paroxysmal nocturnal hemoglobinuria.

Although the American Society of Hematology’s Choosing Wisely Campaign recommends that thrombophilia testing not to be conducted in adult patients with VTE that occurs in the setting of major transient risk factors (surgery, trauma, or prolonged immobility) [14], clinicians continue to order these tests in unselected patients in an incomplete and fragmented manner following an acute episode of a provoked VTE or while on anticoagulation therapy [15]. Thrombophilia testing is influenced by a number of biological and analytical variables including acute inflammatory states or concurrent anticoagulation. Performing such testing at these particular times may confound resultant laboratory values, often leading to false positive test results [15]. Moreover, repeat testing to confirm initial abnormal results is frequently not conducted. This often results in patients carrying a wrong diagnosis leading to long-term anticoagulation with increased bleeding risk and healthcare costs [15].

Considering the lack of evidence supporting the use of these tests in the acute setting, we conducted a retrospective analysis of ordering practices at our academic teaching institutions. Based on the findings from this analysis, we implemented thrombophilia-testing guidelines and subsequently reevaluated ordering practices 22 months later.

## Materials and Methods

This is a retrospective observational study of consecutive unselected patients undergoing thrombophilia testing during the months of October and November 2009 at two teaching hospitals of the University of Texas Southwestern (UTSW) Medical Center in Dallas. Informed consent for the use of each patient’s data was not sought due to the retrospective nature of this study. All patient data was de-identified and anonymized prior to analysis. The UTSW institutional review board approved the study.

Clinical information extracted from the electronic medical record (EMR) included the following: age, sex, thrombosis type and location, indication and timing of testing, comprehensiveness of the tests, anticoagulation therapy at the time of testing, and if abnormal test results were repeated to confirm a diagnosis. Tests included were lupus anticoagulant (LA), anti-cardiolipin (aCL), anti-β2 glycoprotein I (aβ2GPI), anti-phosphatidylserine (aPS), anti-prothrombin (aPT), activated protein C resistance (APCR), FVL, PGM, PC activity, PS activity, AT activity, and FVIII activity. Indications for thrombophilia testing were allocated among nine categories that included: 1) unprovoked venous thrombosis, 2) unprovoked arterial thrombosis, 3) provoked venous thrombosis (risk factors included: immobilization, surgery, trauma, and malignancy prior to or at the time of the thrombotic event), 4) provoked arterial thrombosis (risk factors such as hypertension, dyslipidemia, diabetes mellitus, and known atherosclerotic disease), 5) pregnancy morbidity (including pre-eclampsia and intrauterine growth retardation), 6) recurrent pregnancy losses (≥3), 7) pregnancy losses (<3), 8) others (testing ordered without a prior thrombotic event or adverse pregnancy outcome), and 9) unknown (when there was insufficient data to establish an indication). The timing of thrombophilia testing after the initial presentation was categorized as: within the first week, between the 2^nd^ and 12th week, and beyond 12 weeks. Thrombophilia workup was defined as complete if it comprised of AT, PC, PS, APCR, FVL, PGM, FVIII, LA, aCL, and aβ2GPI.

Data was recorded using Microsoft Access 2003 and analyzed using Microsoft Excel 2003. Where applicable, the Z-test for comparison of proportions was used to assess the statistical significance of observations.

## Results

Of the 173 patients, 125 (72%) were females. The median age was 44 (range 19–83) years. The largest ethnicities represented were White (38%), Hispanic (30%), and Black (28%).

The indications for thrombophilia testing are summarized in Table 1. Fifty-one (29%) patients had testing performed without a documented thrombotic event or pregnancy morbidity. Of these 51 patients, the most common indications for testing were a history of connective tissue disorder or positive serologic tests for an autoimmune condition (21 patients), unconfirmed thrombosis (10 patients), and atypical antiphospholipid syndrome manifestations (8 patients).

**Table 1:** Indications for Thrombophilia Testing.

Indication	Frequency
Unprovoked venous thrombosis	24 (14%)
Provoked venous thrombosis	31 (18%)
Unconfirmed thrombosis	10 (6%)
Unprovoked arterial occlusion	20 (12%)
Provoked arterial occlusion	23 (13%)
Recurrent pregnancy loss	14 (8%)
1–2 pregnancy loss	4 (2%)
Pregnancy morbidity	3 (2%)
History of Connective Tissue Disease or Positive Serologic Tests	21 (12%)
Atypical Antiphospholipid Syndrome Manifestations	8 (5%)
Atypical Thrombosis^a^	6 (3%)
Miscellaneous^b^	6 (3%)
Unknown	3 (2%)
Total	173

^**a**^Includes ischemic colitis, optic neuropathy, livedo reticularis, vasculopathy, leg ulcer, necrotic digits.

^b^Includes infertility, coagulopathy, FVIII inhibitor, easy bruising, rash, and cochlear hydrops.

Of the 173 patients, 119 (69%) had a known thrombotic event(s) or pregnancy loss prior to testing, with 58 of the 173 (34%) patients possessing an indication that was considered appropriate. Appropriate indications were defined as an unprovoked thrombosis (24 venous and 20 arterial) or ≥ 3 pregnancy losses (14 patients). Sixty-one out of 173 patients (35%) were tested when alternative etiologies of thrombosis (31 provoked venous and 23 arterial) were present, and 7 had unqualified pregnancy morbidity or pregnancy loss < 3.

Sixty-one out of 119 (51%) patients with known events were tested immediately after the event (<1 week) when the results could be affected by acute phase reaction and/or anticoagulation therapy. Thirty-eight out of 119 patients (32%) were tested beyond 3 months. The remaining 20 patients (17%) were tested between 1 week and 3 months.

**Fig 1:**
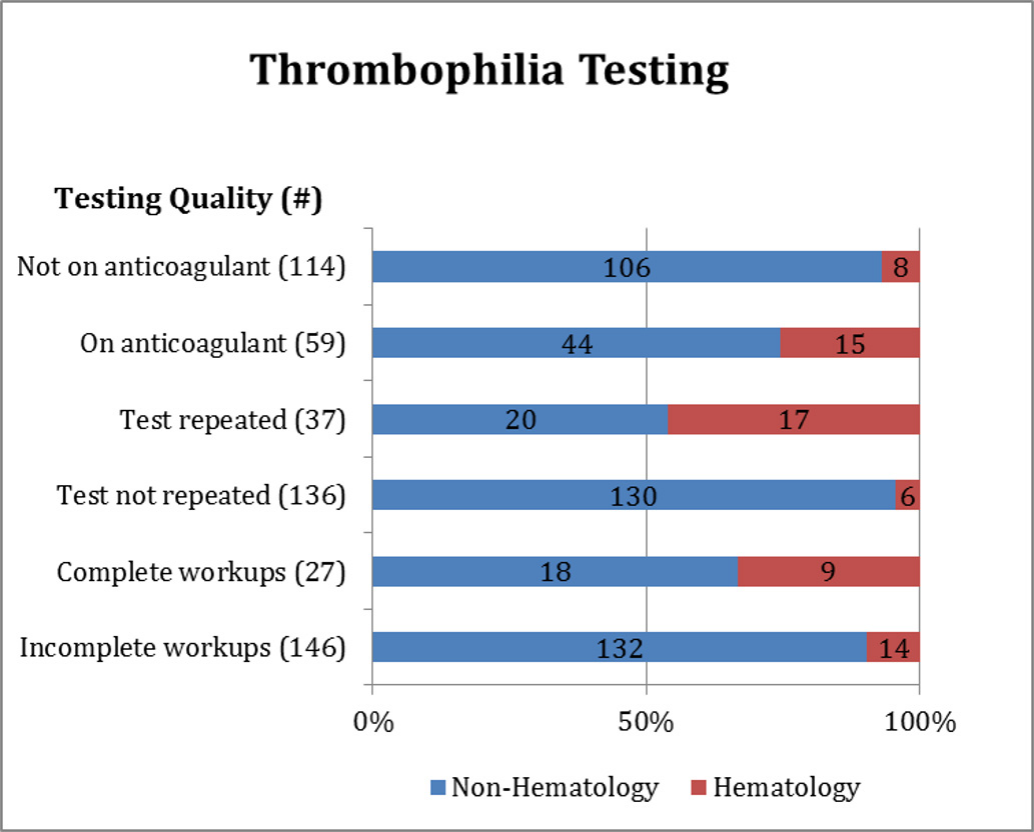
Characteristics of Ordering Practices Prior to Implementation of Guidelines.

Comprehensive testing was performed in only 27 out of the 173 (15.6%) patients (Fig 1). The most commonly ordered tests were LA, aCL, aβ2GPI, and aPS. Abnormal results were found in 79 of the 173 patients (46%) (Table 2). Deficiencies of PC (18/84) and PS (17/84) were identified at a greater frequency (41.6%) compared to FVL (7/69) or PGM (1/63) (11.5%). Initial abnormal test results were repeated in 36 of the 79 patients (46%). Reproducible abnormalities were demonstrated in 24 of the 36 patients (67%) although many of them were retested within 7 days of the first test (Table 2 and Fig 1).

**Table 2:** Thrombophilia Tests Ordered and Results.

Tests	Total Tests (n)	Positive Tests(n)	Repeated Tests (n)	Confirmed Tests (n)
LA	108	17	8	3
aCL	145	36	15	6
aβ_2_GPI	114	28	12	8
aPS	113	20	7	4
aPT	37	3	3	3
APCR	37	4	0	0
FVL	69	7	2	2
PGM	63	1	0	0
PC	84	18	3	1
PS	84	17	3	3
AT	77	3	1	1
FVIII	38	8	5	3

79/173 tested positive for a thrombophilia test; 36/79 had abnormal tests repeated for confirmation; 24/36 had confirmed abnormal results, however, most were performed during hospital stay, thus likely to be false positive.

LA, lupus anticoagulant; aCL, anti-cardiolipin; aβ_2_GPI, anti-beta2 Glycoprotein I; aPS, anti-phosphatidylserine; aPT, anti-prothrombin; APCR, activated protein C Resistance; FVL, Factor V Leiden; PGM, Prothrombin Gene Mutation 20210A; PC, protein C activity; PS, protein S activity; AT, Antithrombin; FVIII, Factor VIII

Fifty-nine out of 173 (34%) underwent testing while on heparin, direct thrombin inhibitors, or vitamin K antagonists for the treatment of an acute thrombosis (Fig 1). Hematologists were more likely to order comprehensive tests and to repeat thrombophilia testing to confirm or refute a diagnosis when compared to the ordering practices of non-hematologists.

A Laboratory Advisory Committee (LAC), composed of representative physicians from all clinical specialties, routinely evaluates ordering practices at our institution. The above findings were presented to the LAC, prompting implementation of local guidelines to improve test utilization and patient care. The committee-approved guidelines for thrombophilia testing are provided in Table 3. The primary recommendation of the committee was to “do not perform thrombophilia testing of patients admitted with an acute VTE, arterial thrombosis, or patients diagnosed with a thrombotic event during their hospital stay”. As per guidelines, these patients were to be investigated in the outpatient setting if they met criteria (age, unprovoked event, etc.) after a minimum of 2 weeks following discontinuation of any oral anticoagulation therapy. Any thrombophilia work up ordered for inpatients was to be investigated by the transfusion medicine hemostasis service. The transfusion medicine hemostasis service would communicate with ordering clinicians if the request did not meet established guidelines to cancel testing that was deemed inappropriate. At this stage, thrombophilia work-ups for unexplained ischemic strokes and to aid the diagnosis of systemic lupus erythematosus could be ordered for inpatients.

**Table 3:** Thrombophilia Testing Panels in the outpatient setting.

Panel	Tests
VTE^a^ or pregnancy loss^b^	1. APLS testing (LA, aCL, aβ_2_GPI); 2. APCR first and if positive, reflexed for FVL; 3 PGM; 4. AT activity, PC activity, PS activity, PS total and free antigen; 5. FVIII
Arterial occlusion not entirely explained by atherosclerosis or vascular injury^c^	1. APLS testing (LA, aCL, aβ_2_GPI); 2. FVIII
Second tier testing after Hemostasis consult	1. JAK2 mutation to rule out myeloproliferative disorders; 2. Flow cytometry for glycosylphosphatidylinositol-anchored surface antigens—to rule out PNH

APLS, antiphospholipid syndrome; LA, Lupus Anticoagulant; aCL, Anti-Cardiolipin; aβ_2_GPI, Anti-Beta2 Glycoprotein I; APC-R, Activated Protein C-Resistance; FVL, Factor V Leiden Gene Mutation; PGM, Prothrombin Gene Mutation 20210A; AT, antithrombin; PC, protein C; PS, protein S; FVIII, factor VIII;; JAK2, Janus Kinase 2; PNH, Paroxysmal Nocturnal Hemoglobinuria.

^a^Recommended only in patients < 55 years.

^b^ Includes ≥ 3 or more recurrent pregnancy losses.

^c^ Recommended only in patients < 40 years.

Twenty-two months following implementation of thrombophilia testing guidelines, an audit of ordering practices of inpatients over a 6-month period (January 1, 2015 and June 30, 2015) was conducted. Thrombophilia testing was ordered on an average of 18 inpatients per month (110 inpatients total over the 6 months) as compared to 87 inpatients per month prior to guideline implementation (79% reduction). Seventy-six tests were ordered per month as compared to 484.5 tests ordered per month prior to guideline implementation (84% reduction). Following electronic consultation with the Transfusion Medicine Hemostasis Service, thrombophilia testing was performed on an average of 5 of the 18 inpatients per month, and 37.5 of the ordered 75 tests were performed per month (Table 4).

**Table 4:** Comparison of thrombophilia testing before and after guideline implementation.

	Before guideline implementation	22 months after guideline implementation	% Reduction
Patients having testing ordered per month	87	18	79%
Patients having testing performed per month	87	5	94%
Tests ordered per month^a^	484.5	76	84%
Tests performed per month^b^	484.5	37.5	92%

^a^Number of tests ordered includes LA, aCL, aβ_2_GPI, aPS, aPT, APCR, FVL, PGM, AT, PC, PS, FVIII.

^b^Prior to guideline implementation, all ordered tests were performed. After guideline implementation, tests were performed only after consultation with the Transfusion Medicine and Hemostasis service.

## Discussion

In this retrospective analysis, only 34% of patients had an appropriate indication for thrombophilia testing, and merely 16% had comprehensive testing performed. Of the 119 patients who had an identifiable thrombotic event or pregnancy morbidity prior to testing, 51% were tested at the time of the event while 34% were on anticoagulation therapy. These factors increase the likelihood of false-positive findings as evidenced by the higher percentage of patients with PC and PS deficiency (approximately 20%) when compared to FVL mutations (10%).

Acute inflammatory states (including thrombotic events) and anticoagulation therapy can often influence analyte testing [16,17]. Consequently, abnormal thrombophilia test results obtained during an acute thrombotic event and on anticoagulation therapy have significant false-positive rates for PS, PC, and AT as supported by our findings of more patients with natural anticoagulant deficiencies as compared to FVL. Similarly false-negative results may be observed due to consumption of antiphospholipid antibodies[16]. It is likely that many of our patients were inappropriately diagnosed with a thrombophilia disorder for which they may have received unwarranted long-term anticoagulation.

In the acute setting, thrombophilia testing does not appear to affect clinical management[18]. Its utility is most directly related to risk-stratification of patients regarding the duration of anticoagulation therapy. However, thrombosis appears to be more influenced by a patient’s non-thrombophilia risk factors including if the patient’s VTE was unprovoked, provoked by a reversible risk factor (i.e. surgery or pregnancy), or provoked by a less transient risk factor (i.e. active malignancy)[19]. In addition, previous studies have demonstrated that thrombophilia testing does not decrease VTE recurrence in patients with an unprovoked VTE[19]. Moreover, in patients with a recurrent unprovoked VTE and a low or moderate bleeding risk, thrombophilia testing is unlikely to affect management as many of these patients receive extended anticoagulation regardless of test results[20].

Thrombophilia testing is expensive with few studies demonstrating their cost-effectiveness [21,22]. Hospitals lose significant revenue on thrombophilia testing due to diagnosis related group (DRG) based payment for inpatients. With a conservative average cost per thrombophilia marker at nearly $100 [23], we approximated $224,200 in wastage over the initial two months of our study. This would amount to a projected wastage of >$1,000,000 over a one year period. However, this monetary value does not account for the cost of unnecessary long-term anticoagulation and its associated complications in patients incorrectly diagnosed with thrombophilia.

Since the management of acute VTE is unaffected by the underlying etiology, the testing should be performed in selected patients 2–4 weeks after discontinuation of anticoagulation therapy. Patients with the following conditions may be tested: unprovoked VTE at a young age (<50 years), a strong family history of VTE, recurrent idiopathic thrombosis, thrombosis at unusual sites, warfarin-induced skin necrosis, or unexplained spontaneous abortions [14,24,25].

Based on the American Society of Hematology Choosing Wisely campaign’s guiding principles of avoiding harm and reducing cost, the Laboratory Advisory Committee established local guidelines for thrombophilia testing (Table 3). Twenty-two months after implementation of our institutional guidelines, we observed an 84% reduction in tests ordered. This suggests that following the implementation of our guidelines, clinicians were cognizant of appropriate thrombophilia ordering practices. However, 18 inpatients still had thrombophilia testing ordered per month; indicating continued clinician education (especially new faculty and house staff) of appropriate ordering practices is still required. After electronic consultation with the Transfusion Medicine and Hemostasis Service, there was an overall 92% reduction in tests performed with an estimated savings of $104,400 per month ($1,252,800 per year). Inappropriate testing can be further reduced by implementing clinical decision-support imbedded within the EMR. These include cascading questions to determine the appropriateness of the test at the time of ordering, best practice alerts (BPAs) [26], or displaying the cost of each test within the EMR[26]. We recently implemented cascading questions in November 2015, and a BPA was applied January 2016. It remains to be seen how ordering practices will change after employment of these alternate measures.

There are inherent limitations to our study due to the retrospective observational design. It was reliant upon review of medical records with most patients not falling under the direct care of the study investigators; thus an intimate knowledge of the subjects included within the study was limited. Conversely, the strengths of this study include the inclusion of consecutive thrombophilia testing orders, a comprehensive and detailed chart review by experts in thrombosis and hemostasis, and the objective collection of data leading to implementation of local guidelines. The reduction in tests orders and tests performed after implementation of local guidelines supports the importance of collective efforts to avoid patient harm and reduce healthcare cost.

## Conclusion

Our study demonstrates that appropriate thrombophilia testing is performed only in a small fraction of patients and results in a significant financial loss. Since thrombophilia defects do not affect the acute management, we propose that a comprehensive thrombophilia work up should be performed only in a select group of patients when they are no longer on anticoagulant therapy. In addition, all abnormal test results should be repeated to confirm the diagnosis to reduce harm by avoiding long term anticoagulation and its bleeding complications. Lastly, implementing best practice guidelines for thrombophilia testing during electronic order placement may improve ordering practices, reduce health care costs, and enhance patient care.

## Supporting Information

S1 Dataset Ordering practices pre-guideline implementation. (XLSM)

S2 Dataset Ordering practices post-guideline implementation. (XLSM)
